# Estimation of Body Height from Head Length among Dental Students of a Dental College

**DOI:** 10.31729/jnma.3751

**Published:** 2018-10-31

**Authors:** Bipana Manandhar, Ritee Shrestha

**Affiliations:** 1 Department of Anatomy, Kantipur Dental College, Basundhara, Kathmandu, Nepal

**Keywords:** *head length*, *height*, *Nepal*, *regression equation*

## Abstract

**Introduction:**

Body height is an important measure of physical identity. Height exhibits a dimensional relationship with various parts of the body. This relationship helps to calculate height from dismembered and mutilated body parts in forensic examinations. As the cranial dimensions are more reliable and precise means of predicting the stature, this study was undertaken to find the relation between head length and height and to derive a regression formula for the estimation of height from head length among dental students.

**Methods:**

This descriptive cross-sectional study was conducted among 150 dental students of age group 18–21 years in the Department of Anatomy, Kantipur Dental College Teaching Hospital and Research Center, Basundhara, Kathmandu. The head length was measured with the help of spreading caliper. Height was measured with the help of a standard height measuring instrument. The head length and the height of the students were measured in centimeters. Data obtained were analyzed to find the correlation between head length and height and to derive a regression equation for the estimation of height.

**Results:**

A significant positive correlation was observed between head length and height (r=0.734, P<0.001). The regression equation for body height and head length including both sexes and all age groups of dental students was found to be y=12.9+8.45x where x is head length and y is body height.

**Conclusions:**

It was observed in the present study that there was a significant positive correlation between height and head length in all the age groups indicating that head length is a reliable indicator in estimation of height.

## INTRODUCTION

Body height is one of the useful anthropometric parameter for individual identification.^1^ Establishing the identity of an individual from mutilated body fragments is an important aspect in natural disasters.^2^ Body height has a definite and proportional biological relationship with each and every part of the human body like head, face, trunk and extremities.^3^ Interrelationship between body height and head length measurements can be used to estimate one from another.^4^

There is no universally applicable formula derived for the estimation of body height from different body parts as the relationship between height and different body parts differ according to race, ethnicity, age and sex. The authors could not find any such study correlating head length with height in context to Nepalese population.

Hence, the present study attempts to find the correlation between head length and body height and estimate body height from head length among dental students of different age group.

## METHODS

This descriptive cross-sectional study was conducted among 150 dental students (107 females and 43 males) of age ranging from 18–21 years in the Department of Anatomy, Kantipur Dental College, Basundhara, Kathmandu, Nepal from February 2018 to June 2018. The study was conducted with the approval of Institutional Review Committee of Kantipur Dental College.

The dental students were selected because of easy access and the students of age group 18–21 years were included as the bone growth usually has ceased after the age of puberty. The students were duly informed about the procedure and the informed consent was taken prior to the procedure. The students with normal cranio-facial skeleton and stature were included in the study. The students with growth disorders, deformities of cranial bone and previous history of craniofacial trauma and surgery were excluded.

Convenience (Non-probability) sampling technique was used to collect data. Sample size was calculated by utilizing following Slovin's^5^ formula based on total number of dental students of Kantipur Dental College.


$$\begin{array}{l} \text{Sample size, n}=\frac{\text{N}}{1+{\text{Ne}}^{2}}\\ =146\\ ∼150 \end{array}$$

where population size (N) = 230, margin of error (e) = 0.05.

Measurements of both head length and the height were recorded in centimetres (cm). The data obtained were analysed statistically using Statistical Package for Social Sciences (SPSS) version 16 to find the correlation coefficient between head length and height and to derive a regression equation for estimating the height from head length. P<0.05 was considered statistically significant.

The regression formula is y = a + bx, where b stands for slope, a stand for intercept/constant, x stands for mean head length and y stands for height.

## RESULTS

The total number of dental students who participated in the study was 150 out of which 107 (71.33%) were females and 43 (28.66%) were males. They were grouped in different age group of 18–21 years.

**Table 1.** Mean and standard deviation of height and head length.

Age group (years)	Frequency n (%)	Sex	Mean±SD of height (cm)	Mean±SD of head length (cm)
18–21	43 (28.66)	Male	169.45±7.06	18.30±0.85
	107 (71.33)	Female	160.27±10.38	17.50±0.81
	150	Total	162.90±10.40	17.73±0.90
18	5 (22.72)	Male	173.33±3.63	18.46±0.77
	17 (77.27)	Female	164.37±9.26	17.67±0.77
	22	Total	164.62±12.64	17.73±1.08
19	7 (13.20)	Male	166.02±6.54	18.15±0.99
	46 (86.79)	Female	158.84±12.05	17.28±0.82
	53	Total	159.79±11.68	17.39±0.88
20	16 (35.55)	Male	165.30±7.49	17.89±0.84
	29 (64.44)	Female	161.36±6.87	17.79±0.68
	45	Total	162.76±7.26	17.82±0.73
	15 (50)	Male		
21	15 (50)	Female		
	30	Total		

The frequency, mean and standard deviation of height and head length of all the dental students were grouped according to age and gender is presented (Table 1). The mean and standard deviation of height and head length of all the students was found to be 162.90±10.40 cm and 17.73±0.90 cm respectively. The mean height and head length were measured to be larger in males than in females (Table1).

**Table 2.** Correlation coefficient and statistical significance.

Age group (years)	Sex	Correlation coefficient (r)	P
18–21	Male	0.706	<0.001
	Female	0.690	
	Total	0.734	
18	Male	0.458	<0.001
	Female	0.651	
	Total	0.830	
19	Male	0.876	<0.001
	Female	0.655	
	Total	0.675	
20	Male	0.700	<0.001
	Female	0.648	
	Total	0.661	
21	Male	0.187	<0.001
	Female	0.570	
	Total	0.741	

**Figure 1.**
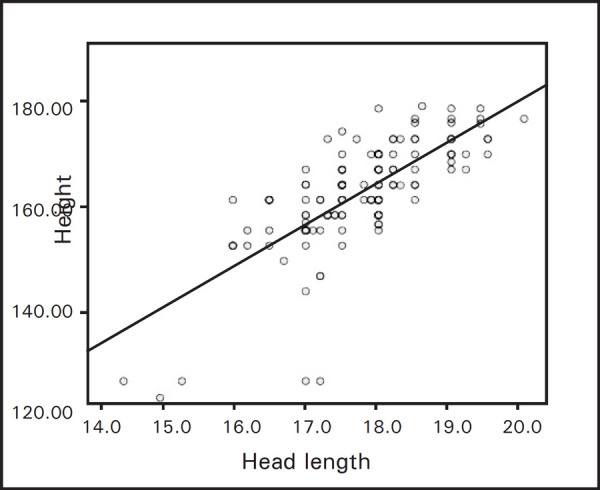
Correlation of head length with height.

The head length showed a positive and significant correlation with height in all the age groups (Table 2, Figure 1).

Estimated height was calculated by using a regression equation, y = a + bx by putting the values of regression constant (a) and regression coefficient (b) from Table 3 and the value of mean head length (x) from Table 1. The measured and estimated height was found to be nearly equal in individual sex of all age groups (Table 3).

**Table 3.** Estimation of height from regression equation.

Age (years)	Sex	Regression constant (a)	Regression coefficient (b)	Measured height (cm)	Estimated height (cm)
18–21	Male	62.52	5.84	169.45	169.39
	Female	7.09	8.74	160.27	160.04
	Total	12.90	8.45	162.90	162.71
18	Male	135.47	2.05	173.33	173.31
	Female	26.89	7.78	164.37	164.36
	Total	-7.13	9.68	164.62	164.49
19	Male	61.61	5.75	166.02	165.97
	Female	-7.52	9.62	158.84	158.71
	Total	4.97	8.89	159.79	159.56
	Male	54.56	6.81	165.30	165.12
20	Female	45.50	6.51	161.36	161.31
	Total	46.72	6.50	162.76	162.55
21	Male	153.75	1.08	174.19	174.02
	Female	37.56	6.97	160.52	160.51
	Total	22.34	7.96	167.36	167.21

## DISCUSSION

Many researchers have conducted studies to estimate height from different body parts. Some researchers have used cranio-facial dimensions and some have used arm measurements6 for estimation of height. This study was undertaken to estimate height from head length by using the regression equation. Dimensional relationships between the body segments and the whole body have been of interest to artists, scientists, anatomists, anthropologists and medicolegistics for long time.^7^ It is worthy to note that there are various factors such as genetic, nutrition, geographical location, physical activity and various races which affect the anthropometric data.^8^

The present study found that head length had a strong and significant positive correlation with the height and there was no significant difference observed in estimated and measured height in dental students of all age groups.^9^ Our study with correlation coefficient of head length with height (r = 0.73) found similarity with the studies carried out in medical students belonging to various regions of Gujarat, India (r = 0.53),^9^ male medical students in Uttar Pradesh, India (r= 0.74),^10^ and medical students belonging to Punjab, India (r = 0.52).^11^ This study was consistent with the study conducted on young adults of Efik and Ibibo, two different ethnic groups of Nigeria^12^ in which a significant correlation between body height and head circumference, length and width was observed.

Our study was in accordance with a cross-sectional study among medical students in Belgaum, India^13^ in which head length from nasion to inion showed a positive correlation with stature for male, female and combined with correlation coefficient of 0.507 in males and 0.440 in females and P<0.001. The present study found resemblance with a study among medical students of cosmopolitan origin of Uttar Pradesh, India^14^ which showed a significant positive correlation of height with both head length and head breadth. Our study revealed that cranial dimension, head length is a statistically significant predictor of height which was found similar with the study conducted on 60 patients in Ghaziabad, India^15^ and on 200 medical students at S.M.S. Medical College, Jaipur, Rajasthan.^16^

The present study with correlation coefficient (r = 0.73) was dissimilar with the study conducted on individuals aged 18–25 years in Ramaiah Medical College, Bengaluru, India^17^ in which a weak positive correlation was observed between height and head length (r=0.27). Our study was not in accordance with the study carried out on Bangladeshi Garo adult females^18^ with correlation coefficient (r = -0.02) in which head length did not reach statistically significant level with their height. This study was in contrast with the study conducted in female medical students of Uttar Pradesh, India^19^ in which head length was observed to be of little importance for estimation of height.

Our study was not in consistent with the study conducted on Indo-Mauritian population in Mauritius^20^ in which accurate stature estimation was not found possible from cephalo-facial dimensions. Our study found no similarity with a research conducted on 100 healthy individuals in Mumbai, India^20^ which did not show the positive correlation of head dimensions with stature.

In the present study, only 150 students with age group 18–21 years were included and ethnic or regional specific regression formulae was also not derived so further studies with larger samples including individuals of age above 21 years of different ethnic groups of Nepal, could be conducted for more better result. Applicability of anthropometric measurements of this study in living may practically differ in deceased individuals. The present study is a preliminary one conducted in Nepal hence need to be followed up by other studies to address the above limitations.

## CONCLUSIONS

The present study showed a positive significant correlation between height and head length indicating that the regression equation from head length can be used as a reliable indicator in prediction of height. The prediction of height from incomplete and decomposed cranial remains is essential in establishing the identity of unknown individuals in incident of murders, accidents or natural disasters. If one of the parameter is known the other can be known by applying the regression equation when the prediction of the height from incomplete and decomposed cranial remains is essential in forensic and anthropology sciences.
