# ERRATUM of Issue 213 JNMA 2018

**Published:** 2019-04-30

The online version of the “Estimation of body height from head length among dental students of a dental college” (Manandhar B, Shrestha R. Estimation of body height from head length among dental students of a dental college. J Nepal Med Assoc. 2018 Sep–Oct;56 (213):861–5) has been updated according to the respective authors' reply published in JNMA 216 Issue.

The following lines were found to be missing in methods section below sample size calculation:

The head length was measured with the help of spreading caliper from glabella to inion when the student was sitting in a chair in a relaxed state. Glabella is the most prominent point on the frontal bone above the root of the nose. Inion is the most prominent point on external occipital protuberance on head in mid-sagittal plane. Body height was measured with the help of a standard height measuring instrument as the vertical distance from vertex to the floor with the student standing barefoot, erect, on an even floor in the Frankfurt's Horizontal Plane. Vertex is the highest point on the head in mid-sagittal plane.

